# Do Antiretroviral Drugs Protect From Multiple Sclerosis by Inhibiting Expression of MS-Associated Retrovirus?

**DOI:** 10.3389/fimmu.2018.03092

**Published:** 2019-01-22

**Authors:** Elena Morandi, Radu Tanasescu, Rachael E. Tarlinton, Dumitru Constantin-Teodosiu, Bruno Gran

**Affiliations:** ^1^Clinical Neurology Research Group, Division of Clinical Neuroscience, University of Nottingham School of Medicine, Nottingham, United Kingdom; ^2^Centre de Physiopathologie de Toulouse Purpan, UPS, INSERM, CNRS Université de Toulouse, Toulouse, France; ^3^Department of Neurology, Nottingham University Hospitals NHS Trust, Nottingham, United Kingdom; ^4^Division of Clinical Neurosciences, Department of Neurology, Colentina Hospital, University of Medicine and Pharmacy Carol Davila, Bucharest, Romania; ^5^School of Veterinary Medicine and Science, University of Nottingham, Nottingham, United Kingdom; ^6^School of Life Sciences, School of Medicine, University of Nottingham, Nottingham, United Kingdom

**Keywords:** multiple sclerosis, human endogenous retroviruses, MSRV, HIV, antiretroviral treatments

## Abstract

The expression of human endogenous retroviruses (HERVs) has been associated with Multiple Sclerosis (MS). The MS-related retrovirus (MSRV/HERV-W) has the potential to activate inflammatory immunity, which could promote both susceptibility and progression toward MS. A connection between HERVs and MS is also supported by the observation that people infected with the human immunodeficiency virus (HIV) may have a lower risk of developing MS than the HIV non-infected, healthy population. This may be due to suppression of HERV expression by antiretroviral therapies (ART) used to treat HIV infection. In this pilot study, we compared RNA expression of the envelope gene of MSRV/HERV-W, as well as Toll-like receptors (TLR) 2 and 4, in a small cohort of HIV+ patients with MS patients and healthy controls (HC). An increased expression of MSRV/HERV-W*env* and TLR2 RNA was detected in blood of MS patients compared with HIV patients and HC, while TLR4 was increased in both MS and HIV patients. There was, however, no difference in MSRV/HERV-W*env*, TLR2 and TLR4 expression between ART-treated and -untreated HIV patients. The viral protein Env was expressed mainly by B cells and monocytes, but not by T cells and EBV infection could induce the expression of MSRV/HERV-W*env* in Lymphoblastoid cell lines (LCLs). LCLs were therefore used as an *in vitro* system to test the efficacy of ART in inhibiting the expression of MSRV/HERV-W*env*. Efavirenz (a non-nucleoside reverse transcriptase inhibitor) alone or different combined drugs could reduce MSRV/HERV-W*env* expression *in vitro*. Further, experiments are needed to clarify the potential role of ART in protection from MS.

## Introduction

Multiple Sclerosis (MS) is a demyelinating disease of the central nervous system (CNS). The cause of MS is not known, but genetic linkage studies and the beneficial effect of immunomodulatory treatments indicate that the disease is immune-mediated (1). Activation of autoimmune processes in MS results from the interaction of genetic and environmental factors (which include Herpes viral infections, smoking, and vitamin D deficiency) (2). An additional risk factor that has been identified is the expression of human endogenous retroviruses (HERVs) of the HERV-W family (3, 4).

HERVs are viruses that have integrated into human germ line cells between 30 and 70 million years ago, becoming part of human DNA and being then transmitted through subsequent generations. Indeed, almost 8% of the human genome is constituted of groups of HERVs, ranging in copy number from one to many thousands (5).

It has been reported that endogenous retroviruses can engage the innate immune system, and in particular Toll-like receptors (TLRs) (6). Both *in vitro* and *in vivo* experiments have illustrated the immunopathogenicity induced by MS-associated retrovirus (MSRV/HERV-W) proteins through direct interaction with TLR4 (7–9). Upon TLR4 engagement by HERVs, signaling pathways are activated that lead to secretion of pro-inflammatory cytokines, such as IL-1β, IL-6, and TNF-α (7).

Comorbidity of Human Immunodeficiency Virus (HIV) and MS is very rare (10). Gold et al. examined the association between HIV and MS using an English medical database with a cohort of 21,207 HIV-positive patients and 5,298,496 controls stratified by age, sex, year of first hospital admission, a region of residence, and socioeconomic status. They calculated that the risk rate ratio of developing MS was significantly lower in people infected than in those not infected by HIV (0.38; 95% CI 0.15 to 0.79) (10). The authors discussed two different hypotheses that could explain this inverse correlation. The first is related to the HIV viral infection itself. HIV is an infectious retrovirus that if left untreated causes suppression of the immune system, eventually leading to life-threatening infections and cancers. Primarily, HIV targets the CD4+ lymphocytes cells, which are in their turn thought to be involved in the pathogenesis of MS. The reduction of CD4+ T cells in infected people could, therefore, lower any autoimmune response against the CNS. However, clinical cases where patients have developed MS or CNS demyelinating disorders after HIV infection have been reported (11–13), suggesting thereby that HIV may not protect from MS. Conversely, as HIV-infected MS patients who received Antiretroviral Therapy (ART) had a less severe clinical course of MS (11, 14, 15), this would suggest that if an inverse association between the infection and MS exists, it may in fact be due to the effect of ART on MS. Unfortunately, Gold et al. did not report which patients were taking retroviral treatments, but rather assumed that most of the patients were on ART, as one would expect to be the case for developed countries (10).

Antiretroviral drugs are classified based on the phase of the retroviral life-cycle that each drug targets. Typically, a combination of drugs from different classes are used to optimize their efficacy in the treatment of HIV infection [termed ART, or combination anti-retroviral therapy (cART)]. These antiretroviral therapies act not only against HIV but probably also inhibit endogenous retroviruses, which thereby could potentially prevent the development of MS. In line with this contention, a phase II clinical trial (INSPIRE) studying the effect of the integrase (enzyme that inserts the viral genome into the DNA of the host cell) inhibitor Raltegravir on relapsing-remitting (RR)-MS patients has been completed. Unfortunately, this trial did not show any impact of the drug on MS inflammatory activity detected by MRI (16). However, as HERVs are already integrated into the genome, they may not be affected by an integrase inhibitor.

In the current study, we aimed to test the hypothesis that ART can reduce the expression of HERVs. A small cohort of HIV+ patients who were or were not on ART was recruited to study *in vivo* the effect of antiretroviral drugs on the expression of human MSRV/HERV-W. In parallel, the same classes of the drug were used to test their efficacy in MSRV/HERV-W inhibition *in vitro*, treating Lymphoblastoid cell lines (LCL).

## Materials and Methods

### Human Blood Samples

Blood samples from patients attending Nottingham University Hospitals NHS Trust were collected in PAXgene Blood RNA tubes (Qiagen) and directly stored at −80°C and later used for gene expression analysis. MS diagnosis was established according to McDonald's criteria (17), and HIV infection was certified at the Microbiology Department at the same hospital. All patients and HCs signed informed consent for which ethical approval was obtained. Patients and HC age, gender and clinical status are presented in Table 1.

**Table 1:** Information on subjects in the study.

**Subject**	**Age**	**Gender**	**Ethnicity**
**(A) INFORMATION ON HC**			
HC 1	32	M	Caucasian
HC 2	44	F	Caucasian
HC 3	47	F	Caucasian
HC 4	38	F	Caucasian
HC 5	27	M	Caucasian
HC 6	54	M	Caucasian
HC 7	31	F	Arabic
HC 8	30	M	Caucasian
HC 9	51	F	Jamaican
HC 10	42	M	Caucasian
HC 11	27	M	Caucasian
HC 12	44	M
HC 13	60	F	Caucasian
HC 14	44	F	Caucasian
*HC 15*	*55*	*F*	Caucasian
HC 16	28	M	Caucasian
HC 17	60	M	Caucasian
HC 18	53	F	Caucasian
HC 19	59		Caucasian
HC 20	31	M	Indian
HC 21	27	M	Caucasian
HC 22	44	F	Caucasian

**Table:** 

**Subject**	**Age**	**Gender**	**Ethnicity**	**Disease course**	**Current DMT**	**Disease duration (years)**
**(B) INFORMATION ON MS PATIENTS**						
MS 1	41	F	Caucasian	RR	no	5
MS 2	56	F	Caucasian		no	8
MS 3	38	F	Caucasian	RR	no	3
MS 4	54	M	Caucasian	RR	no	6
MS 5	55	F		SP	no
MS 6	46	F		SP	no
MS 7	44	F		RR	no
MS 8	46	F		RR	no
MS 9	52	F		SP	no
MS 10	21	M		RR	no
MS 11	61	F		SP	no
MS 12	46	F		SP	no
MS 13	51	F	Caucasian	RR	no	1
MS 14	56	F	Caucasian	PP	no	8
MS 15	56	F			no
MS 16	52	F	Caucasian	SP	no	6
MS 17	46	F	Caucasian		no
MS 18	48	F	Caucasian		no
MS 19	49	M	Caucasian	RR	β-interf	1
MS 20	45	F	Caucasian	RR	no
MS 21	41	F	Caucasian		no	2
MS 22	42	M	Caucasian	RR	β-interf	1

**Table:** 

**Subject**	**Age**	**Gender**	**Ethnicity**	**Art**	**Duration on art**	**Viral load**
**(C) INFORMATION ON HIV PATIENTS**						
HIV 1	46	M	Greek	no	–	30,010
HIV 2	31	M	Jamaican	no	–	608
HIV 3	48	M	White British	Tdf, FTC, DRV, Rit	4 years	<40
HIV 4	49	F	White British	ABC, 3TC, EFZ	13 years	<40
HIV 5	34	F	Filipino	no	–	106,361
HIV 6	42	M	White British	Tdf, FTC, EFZ	3 years	<40
HIV 7	57	M	White British	Tdf, FTC, EFZ	9 years	<40
HIV 8	47	F	White British	AZT, 3TC, EFZ	9 years	<40
HIV 9	44	M	White British	Tdf, FTC, DRV, Rit	12 years	<40

*RR, relapsing-remitting; PP, primary-progressive; SP, secondary-progressive*.

ARTs (antiretroviral treatments):

***NRTIs**: ABC: Abacavir, AZT: Zidovudine, 3TC: Lamivudine, FTC: Emtricitabine*.

***NtRTI:** Tdf: Tenofovir disoproxil*.

***NNRTIs**: EFZ: Efavirenz*.

***PIs:** DRV: Darunavir, Rit: Ritonavir*.

*Subject HC 15 (italics) was used as reference for relative quantification of MSRV/HERV-Wenv, TLR4 and TLR2 gene expression*.

### RNA Extraction and cDNA Synthesis

Total RNA was purified using the PAXgene Blood RNA kit (Qiagen) following the manufacturer's instructions. The RNA was treated with DNase to remove trace amounts of bound DNA. After the wash steps, RNA was extracted in the elution buffer provided with the kit and stored at −80°C. RNA concentration was determined by measuring the absorbance at 260 nm using NanoDrop ND-100 (Thermo Scientific). For making cDNA, 0.5 μg RNA samples, 2 μl Random hexamers and 1 μl dNTPs mix (stock solution 10 mM) (all Promega) were mixed, followed by a 5 min incubation at 65°C for first strand cDNA synthesis. A master mix containing RNase inhibitor (Promega, United Kingdom), DTT (0.1 M) and 5X First-Strand Buffer was added, along with Superscript III RT (220 U/μl) (all from Invitrogen, United Kingdom). Negative controls replacing the RNA template or the RT with DNase/RNase free H_2_O were included. Samples were incubated as followed: 5 min at 95°C, 60 min at 50°C, and 25 min at 70°C. cDNA was stored at −80°C.

### Real-Time RT-PCR

After titration of different cDNA dilutions, 1:2 DNase/RNase free H_2_O dilution was selected. Relative quantification was performed using Hydroxymethylbilane Synthase (HMBS) as housekeeping gene. Nine hundred nanomolar of probe/forward and reverse primer mix of MSRV//HERV-W*env* (designed as reported previously in the literature (18), TLR4, TLR2, and HMBS (TaqMan, Invitrogen) were used. All samples were run in duplicates. RT- PCR reactions were performed using the 7900HT Fast Real-Time PCR system (Applied Biosystems) in 96-well plates. The following incubation protocol was imposed: 10 min at 95°C and 40 cycles of 10 s at 95°C followed by 30 s at 60°C. The mean Ct values of MSRV//HERV-W*env*, TLR4 or TLR2 were normalized compared to the Ct value of HMBS. Calculation of the relative amounts of MSRV//HERV-W*env*, TLR4 or TLR2 was performed using the 2^Δ*ΔCt*^ method with one HC used as a standard (HC_15_, selected for the high amount of RNA concentration). The reference sample HC_15_ was analyzed in all the different plates as an inter-run calibrator. Any change in gene expression between HC, MS and HIV patients compared to HC_15_ was expressed as a fold change using the formula below:

Fold increase for each patient compared to the HC_15_ = 2^Δ*ΔCt*^

Where for each patient:

$$\begin{array}{l} \Delta \Delta Ct=\left[\left(CtGI-CtHMBS\right)-\Delta {CtHC}_{15}\right]\\ \text{GI}=\text{MSRV }env,\text{TLR}4,\text{TLR}2\\ \Delta CtHC_{15}=\Delta Ct\;reference\;gene\left({HC}_{15}\right)\\ =\left({Ct\;GIHC}_{15}-{CtHMBSHC}_{15}\right) \end{array}$$

### Extracellular FC Staining for HERV-W

Peripheral blood mononuclear cells (PBMC) were isolated from blood donors using Ficoll density gradient centrifugation. Surface staining for Pe-Cy7 anti-CD3 (UCHT1), PE anti-CD14 (M5E2), and Pe-Cy5 anti-CD20 (2H7) all from BD was performed on PBMC. HERV-W was detected using primary anti-HERV-WEnv rabbit polyclonal antibody (Allele Biotech) with secondary CF488A goat anti-rabbit antibody (Sigma-Aldrich). A control with only secondary CF488A goat anti-rabbit antibody was included, and Fluorescence minus one (FMO) samples were used to set the gating. Cells were incubated in the dark for 30 min at 4°C. After incubation, cells were washed twice with FACS buffer and centrifuged at 300 g for 6 min. Cells were then fixed with 500 μl Fixation buffer (2% paraformaldehyde, BD). Blue Live/dead marker (ThermoFisher Scientific) was included in the staining to assess the viability. Cells were analyzed by FC using LSRII flow cytometer (BD Biosciences, USA) and FlowJo software (version V10, FlowJo, LLC, United States).

### Activation of B Cells With CpG and Generation of LCL

CD20+ B cells were purified from PBMC by positive selection using a CD20+ cell isolation kit (Miltenyi Biotec) following the manufacturer's instructions. Collected CD20+ and CD20– cells were counted with haemocytometer. Purity of CD20+ cells was assessed using an anti-CD20 Ab by FC. 10^6^ CD20+ isolated B cells were cultured in 400 μl of complete medium [Roswell Park Memorial Institute medium (RPMI) with 10% fetal calf serum (FCS), 100 units penicillin−1 mg/ml streptomycin (pen/strep), 20 mM L-glutamine all from Sigma- Aldrich] with 30 μg/ml of CpG OND 2006 (Invivogen, sequence “TCG TCG TTT TGT CGT TTT GTC GT”) in 48-wells plate for 24 h. Activation of CD20+ cells was assessed using an anti-CD86 Ab by FC.

Five to 10 × 10^6^ of isolated PBMC were centrifuged at 300 g for 10 min. Three ml of supernatant from a B95.8 EBV infected marmoset B cell line (19) (kindly donated by Jill Brooks, Birmingham, UK) was collected from the same batch and centrifuged at 200 g for 5 min. The B95.8 supernatant was filtered through a 0.45 μm filter on the PBMC pellet, and the PBMC mixed with the virus were incubated overnight at 37°C. After centrifugation, the supernatant was discarded, and infected PBMC were re-suspended in complete RPMI + 1 μg/ml Cyclosporine A (Sigma). LCL was established from 6 different healthy donors and was expanded in culture.

### Drug Treatment

Reverse transcriptase inhibitors inhibit HIV reverse transcription, and are divided into Nucleoside Reverse Transcriptase Inhibitors (NRTI), Nucleotide Reverse Transcriptase Inhibitors (NtRTI), and Non-Nucleoside Reverse Transcriptase Inhibitors (NNRTI). NRTI and NtRTI act as competitive substrate inhibitors, while NNRTI inhibits reverse transcriptase by binding to an allosteric site of the enzyme. Integrase inhibitors (II) inhibit the viral enzyme integrase, and protease inhibitors block the viral protease enzyme. Drugs were purchased from Sigma-Aldrich and dissolved in DMSO. One million LCL from different donors were treated with Lamivudine (NRTI), Tenofovir (NtRTI), Daranuvir (PI), Efavirenz (NNRTI) and Raltegravir (II) at concentrations of 10, 1, and 0.1 μM for 5 days. At day 3, the medium was changed, and fresh drugs were added. The range of different doses was chosen based on datasheet indication and previous publications where similar tests *in vitro* were used to test antiretroviral efficacy against HIV (20, 21) or porcine endogenous retroviruses (22).

### Statistical Analysis

The data were presented as medians and interquartile range, and statistical analysis was performed using GraphPad prism v7 as reported in the figure legends.

## Results

### Expression of MSRV/HERV-W in MS and HIV Patients

**Figure 1:**
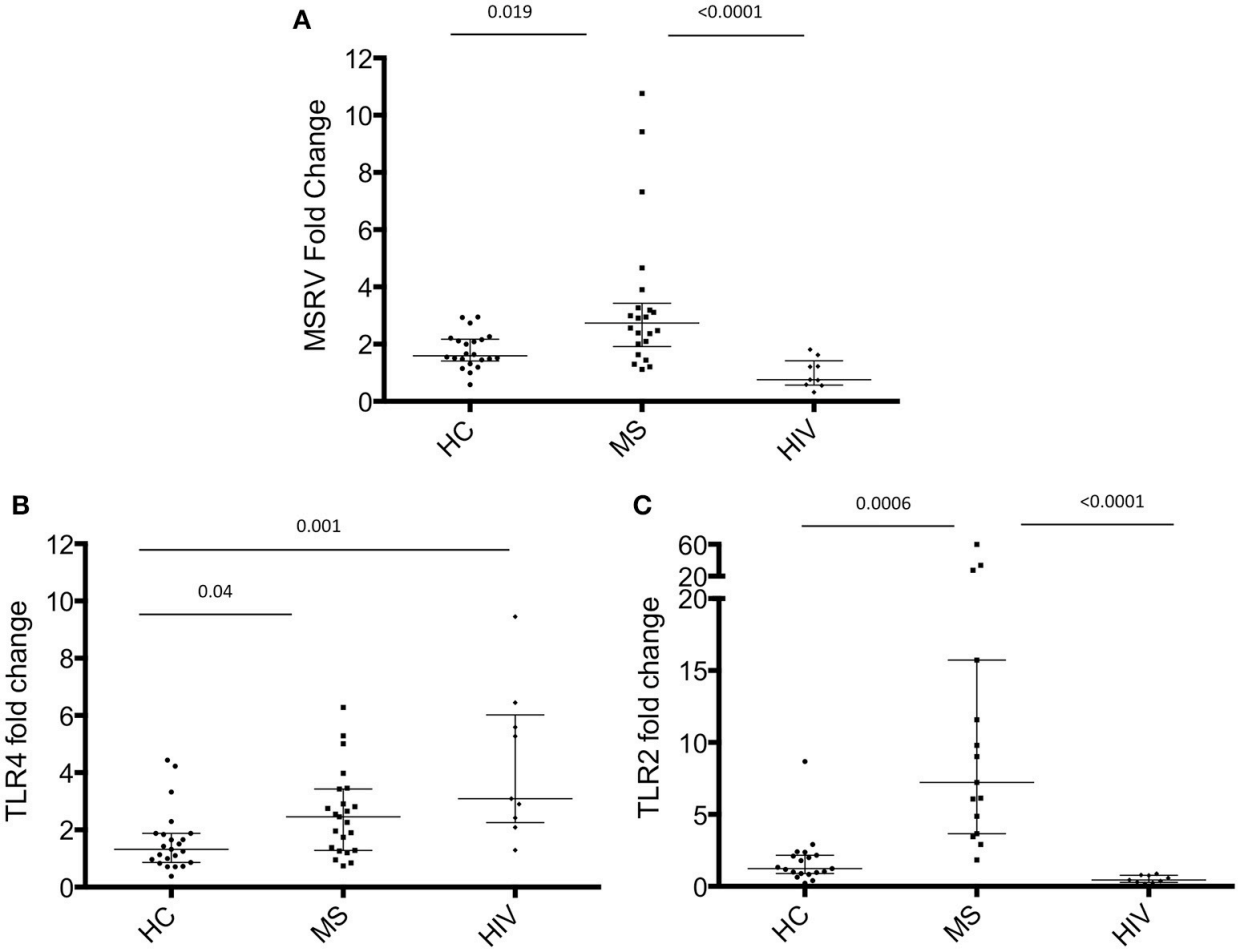
MSRV/HERV-W*env*, TLR4 and TLR2 gene expression in HIV and MS patients and in healthy control (HC). MSRV/HERV-W*env* and TLR4 expression were evaluated by relative quantification RT-PCR using the 2^−Δ*ΔCt*^ method. Fold changes of **(A)** MSRV/HERV-W*env*, **(B)** TLR4, and **(C)** TLR2 expression of each individual sample in each group are illustrated in dots. HIV patients were compared to HC and MS using HC15 as a reference gene. Medians and interquartile range are indicated by bars (HC, *n* = 22, MS, *n* = 22, HIV, *n* = 9; Kruskal-Wallis Test with Dunn's multiple comparison test).

Many studies have been reported in the literature showing the increased expression of MSRV/HERV-W in MS patients (4), but the expression of MSRV/HERV-W in HIV-infected patients has never been investigated until now. In this study, total RNA was extracted from PAXGENE tubes (total blood) of 22 MS patients, 22 HC and 9 HIV-infected patients (Table 1, average age MS = 47.5, HC = 42.8, HIV = 44.2). Relative quantification of MSRV/HERV-W*env*, TLR4 and TLR2 gene expression was assessed with real-time quantitative RT-PCR using the reference gene HMBS and the HC subject number 15 (HC 15, Table 1) as 1. The expression of MSRV/HERV-W*env* in the MS group was significantly higher compared with HC (*p* = 0.019) and HIV (*p* < 0.001) (Figure 1A). MSRV/HERV-W*env* expression was similar in HIV patients and HC.

TLR4 expression was significantly higher in MS and HIV compared to HC (respectively *p* = 0.04 and *p* = 0.001) (Figure 1B), while TLR2 was increased only in MS patients (Figure 1C).

**Figure 2:**
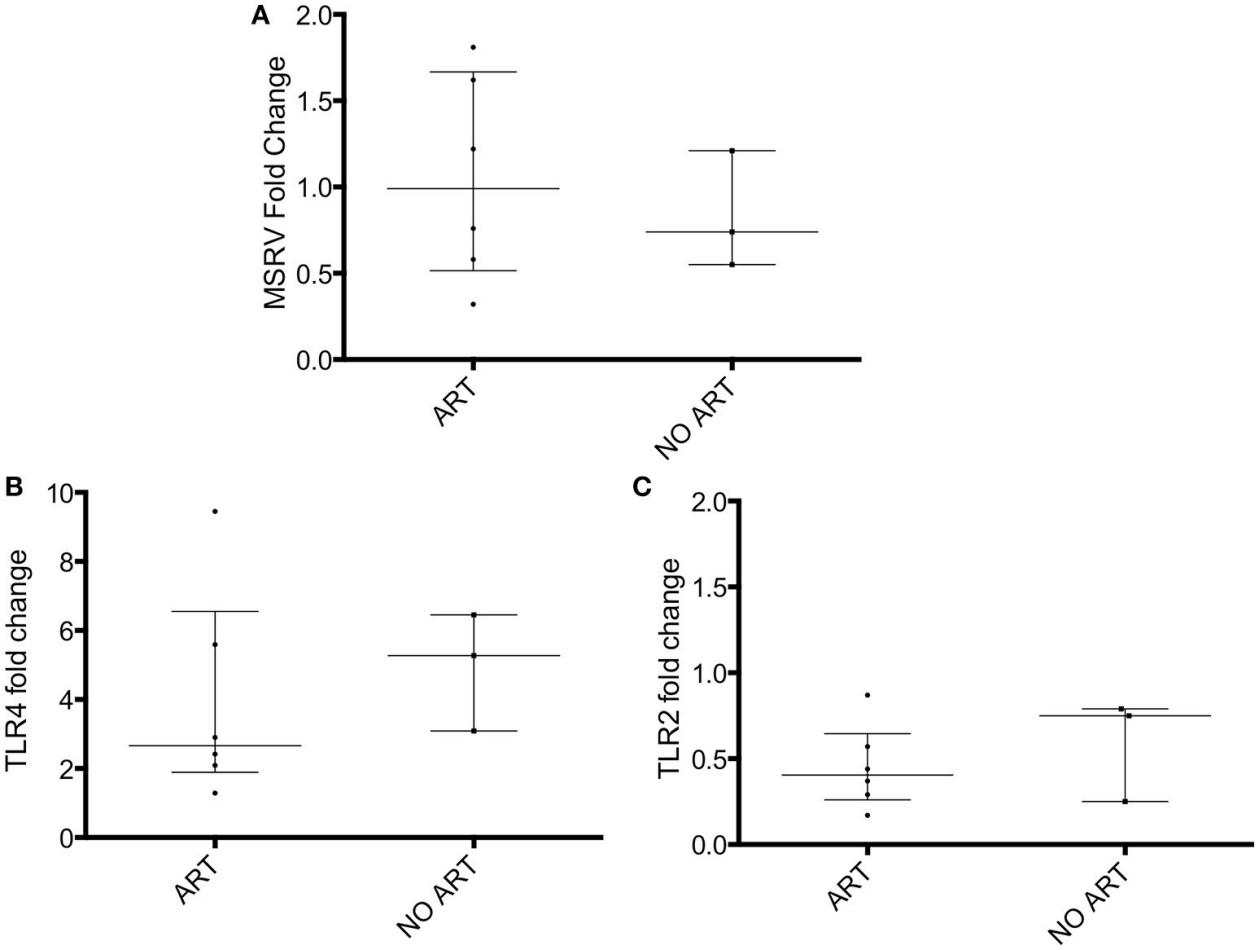
MSRV/HERV-W*env*, TLR4 and TLR2 gene expression in HIV patients categorized in treated (ART) and untreated patients (NO ART). MSRV/HERV-W*env*, TLR4 and TLR2 expression were evaluated by relative quantification RT-PCR using the 2^−Δ*ΔCt*^ method. Fold changes of **(A)** MSRV/HERV-W*env*, **(B)** TLR4, and **(C)** TLR2 expression of 9 HIV patients were categorized in treated (ART) and untreated (NO ART) patients are illustrated in dots. Medians and interquartile range are indicated by bars (ART, *n* = 6, NO ART, *n* = 3; Mann-Whitney Test).

The HIV patients were then grouped into treated with antiretroviral drugs patients (*n* = 6) and untreated patients (*n* = 3; Table 1C). No difference in MSRV/HERV-W*env*, TLR4 and TLR2 expression between the two groups was found (Figure 2).

### Expression of HERV-WEnv Protein in Different Cell Types of MS and HC

**Figure 3:**
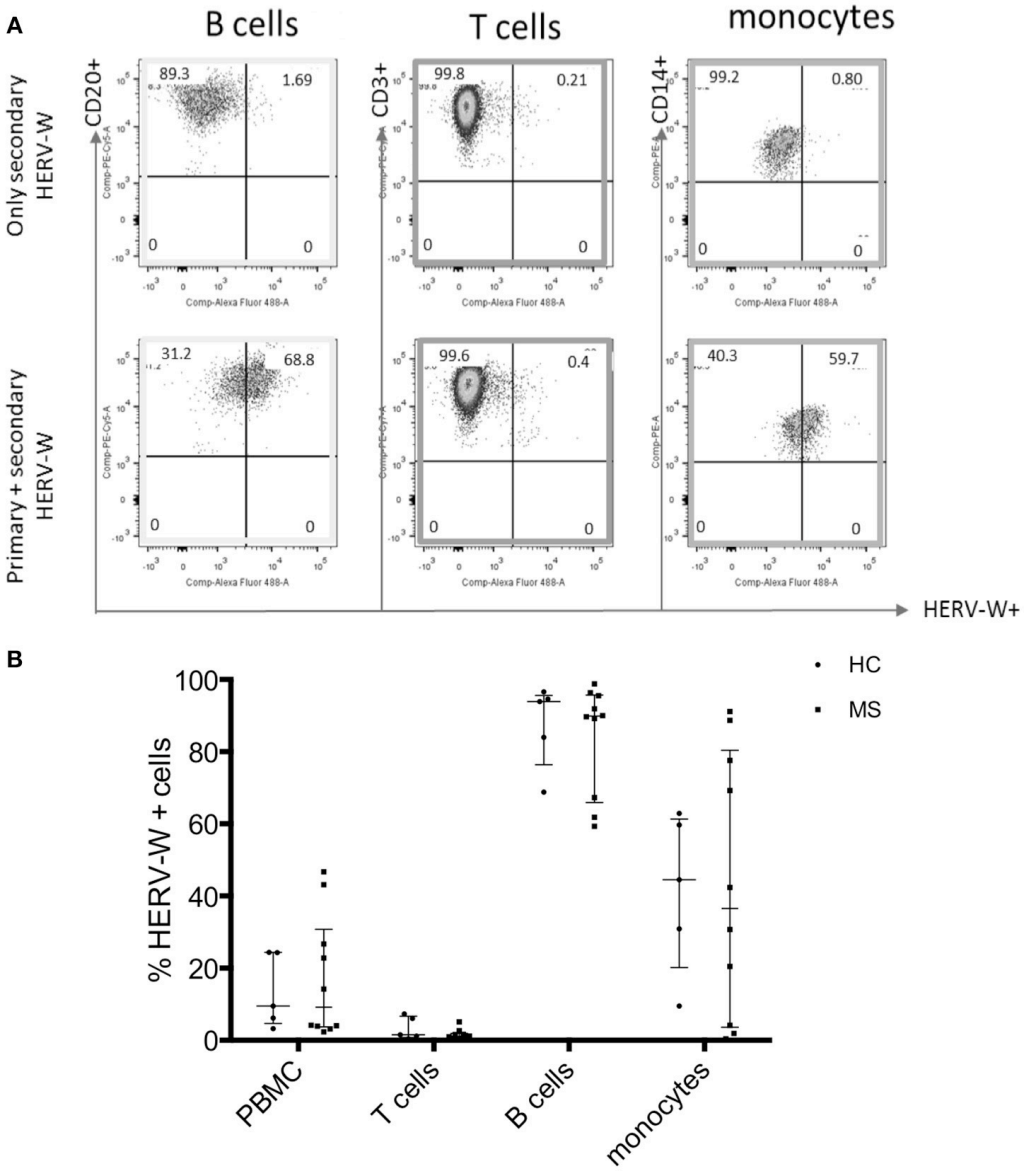
Expression of HERV-WEnv protein in different immune cells in HC and MS by Flow Cytometry. **(A)** Representative dot plot of the expression of HERV-WEnv in gated CD20+ and CD3+ lymphocytes and CD14+ monocytes. The staining with only secondary CF 488A anti-rabbit antibody (without anti-HERV-WEnv primary Ab) was used as negative control. **(B)** Expression of HERV-WEnv was detected in total PBMC, CD3+ T cells, CD20+ B cells and CD14+ monocytes from 10 MS patients and 6 HC. Percentage of positive cells is illustrated in dots. Medians and interquartile range are indicated by bars (n MS = 10, n HC = 6; Mann-Whitney Test).

We next aimed to characterize the cells that express MSRV**/**HERV-W. HERV-W Env can be translated into protein and detected on the surface of cells. HERV-W Env protein expression was detected on the cell surface of immune cells from HCs using a primary anti-HERV-W Env antibody and a CF488A-labeled secondary antibody by Flow Cytometry (23). HERV-W Env was expressed by 20% of PBMC of which mainly by CD20+ B cells and CD14+ monocytes. CD3+ T cells were negative (Figure 3).

Comparison of HERV-W Env protein expression in different cell subtypes (total PBMC, T cells, B cells, and monocytes) derived from 10 MS (6 RR-MS, 1 PP-MS, 3 SP-MS) patients and six age-matched HC, showed no difference in HERV-W Env protein levels (Figure 3B).

**Figure 4:**
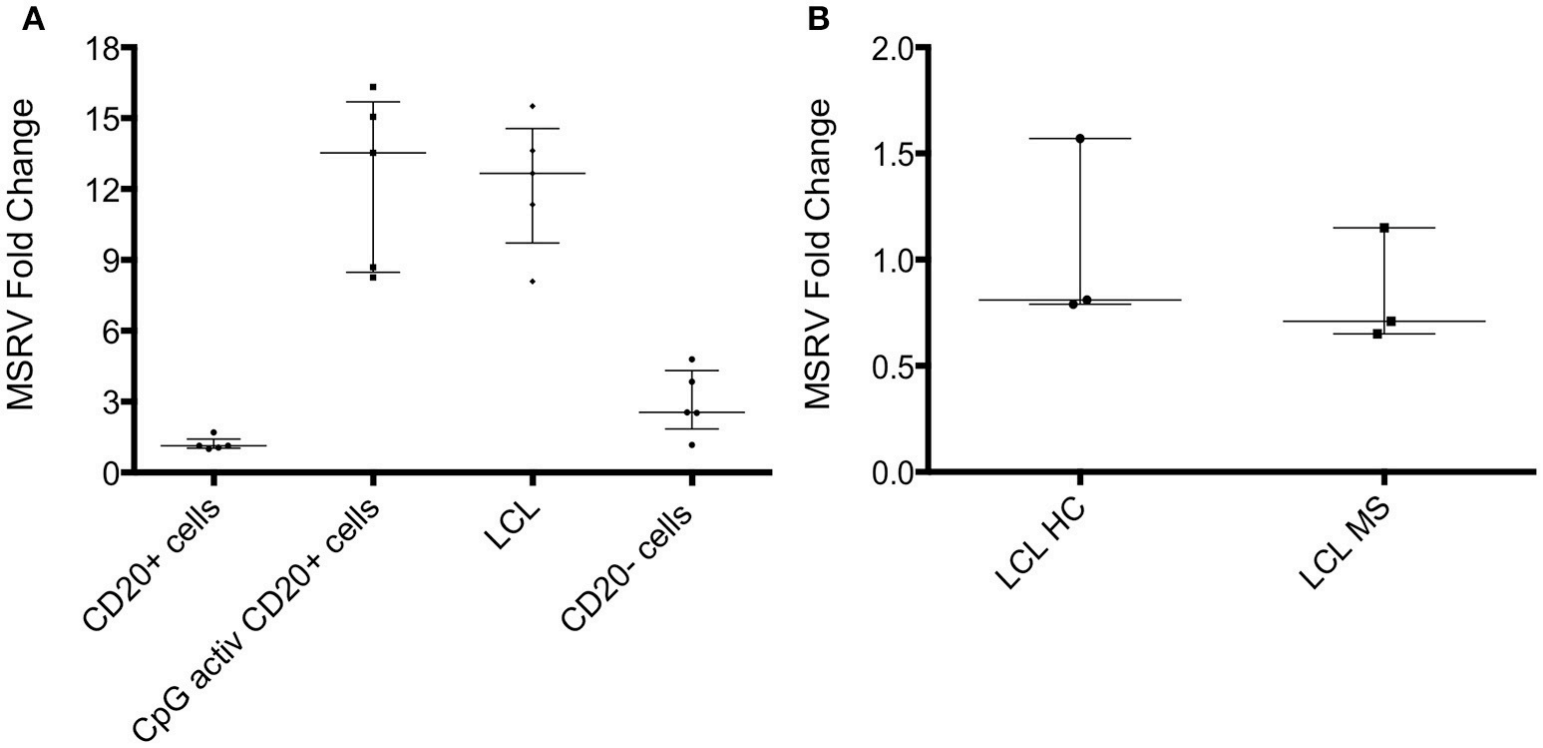
MSRV/HERV-Wenv expression increases in LCL and CpG activated cells. **(A)** MSRV/HERV-Wenv expression was evaluated by relative quantification RT-PCR using the 2-^Δ*ΔCt*^ method. Fold changes of MSRV/HERV-Wenv in isolated CD20+ cells, CpG activated CD20+ cells, LCL (B-lymphoblastoid cell line) and CD20– cells are illustrated in dots. Medians and interquartile range are indicated by bars (n = 5, Kruskal-Wallis Test with Dunn's multiple comparison test). **(B)** MSRV/HERV-Wenv expression was evaluated by relative quantification RT-PCR using the 2^−Δ*ΔCt*^ method. Fold changes of MSRV/HERV-Wenv in LCL from HC and MS patients are illustrated in dots. Medians and interquartile range are indicated by bars (n HC = 3, n MS = 3; Mann-Whitney Test).

EBV is strongly associated with MS (24). *In vitro*, the expression of MSRV/HERV-W genes/proteins is activated by some viruses such as EBV, herpes simplex virus type 1 or by influenza virus (25). One study showed that binding of the EBV surface glycoprotein gp350 could activate the expression of MSRV/HERV-W in cells from blood and brain (23). EBV-immortalized B-lymphoblastoid cell lines (LCL) were generated *in vitro*. LCL represents a tissue culture model for human B cell transformation and virus latency, and they express the genes of the latency III program of EBV. To investigate if EBV could activate MSRV/HERV-W, MSRV/W *env* RNA expression was measured in isolated CD20+ cells, CD20+ cells activated with CpG (TLR9 agonist), LCL and CD20– cells from 5 different HC donors. Quantification of MSRV/HERV-W *env* gene expression relative to the mean of the expression in CD20+ cells (considered as 1) was assessed with real-time RT-PCR. LCL showed 12-fold increase in MSRV/HERV-W expression compared to the uninfected CD20+ cells (Figure 4A). Similarly, CpG activation induced the expression of MSRV/HERV-W *env*.

Relative MSRV/HERV-W *env* RNA expression was measured in LCL from 3 HC and from 3 MS to test if MS patients express more MSRV/HERV-W*env* RNA after EBV infection compared to HC. No difference was detected between LCL from HC and MS by real-time RT-PCR (Figure 4B).

### Expression of MSRV/HERV-W in LCL Treated With Different Antiretroviral Drugs *in vitro*

**Figure 5:**
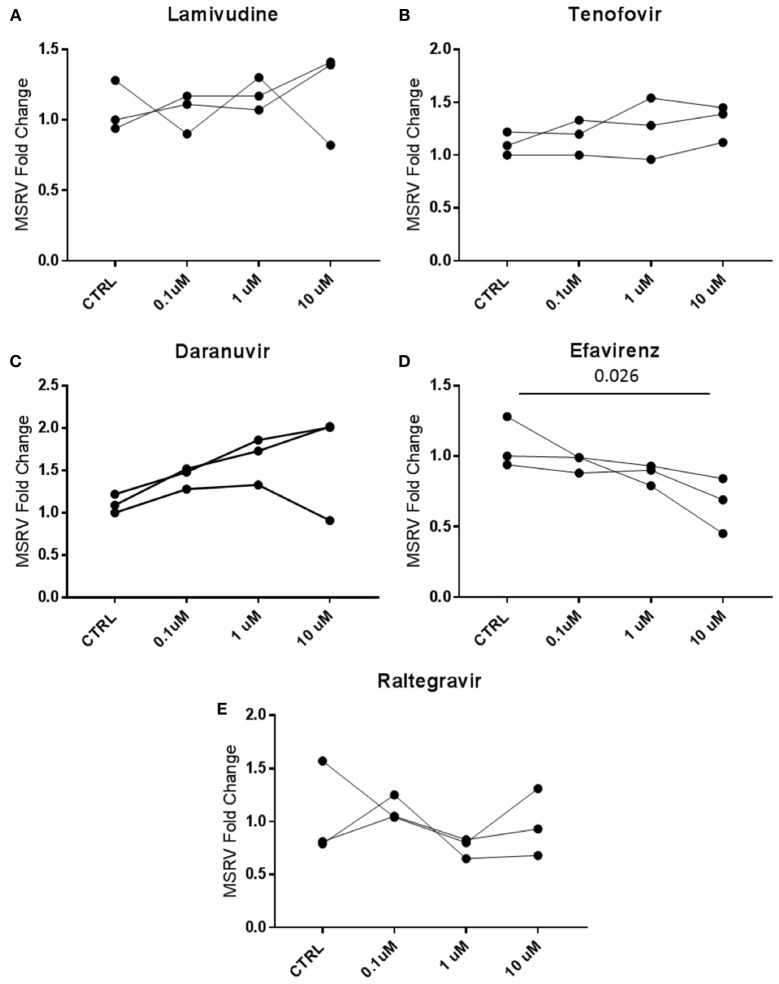
Efavirenz decreases the expression of MSRV/HERV-W*env*. LCL from 3 different healthy donors were treated with 0.1, 1, and 10 μM of **(A)** Lamivudine, **(B)** Tenofovir, **(C)** Daranuvir, **(D)** Efavirenz, and **(E)** Raltegravir for 5 days. CTRL (Controls) represent LCL treated only with DMSO. MSRV/HERV-W*env* expression was evaluated by relative quantification RT-PCR using the 2^−Δ*ΔCt*^ method and is illustrated in dots (*n* = 3; Friedman test).

MSRV/HERV-W*env* expression was then analyzed in cells treated *in vitro* with antiretroviral drugs. MSRV/HERV-W*env* RNA expression was analyzed by RT-PCR in LCL treated with the same classes of drugs that the patients were taking. The drugs used were Lamivudine (NRTI), Tenofovir (NtRTI), Daranuvir (PI), Efavirenz (NNRTI) and Raltegravir (II). LCL from 3 HC subjects were treated with drugs at the concentrations of 10, 1, and 0.1 μM for 5 days (Figure 5). Only Efavirenz (NNRTI) decreased the expression of MSRV/HERV-W*env* at the highest concentration (Figure 5D).

**Figure 6:**
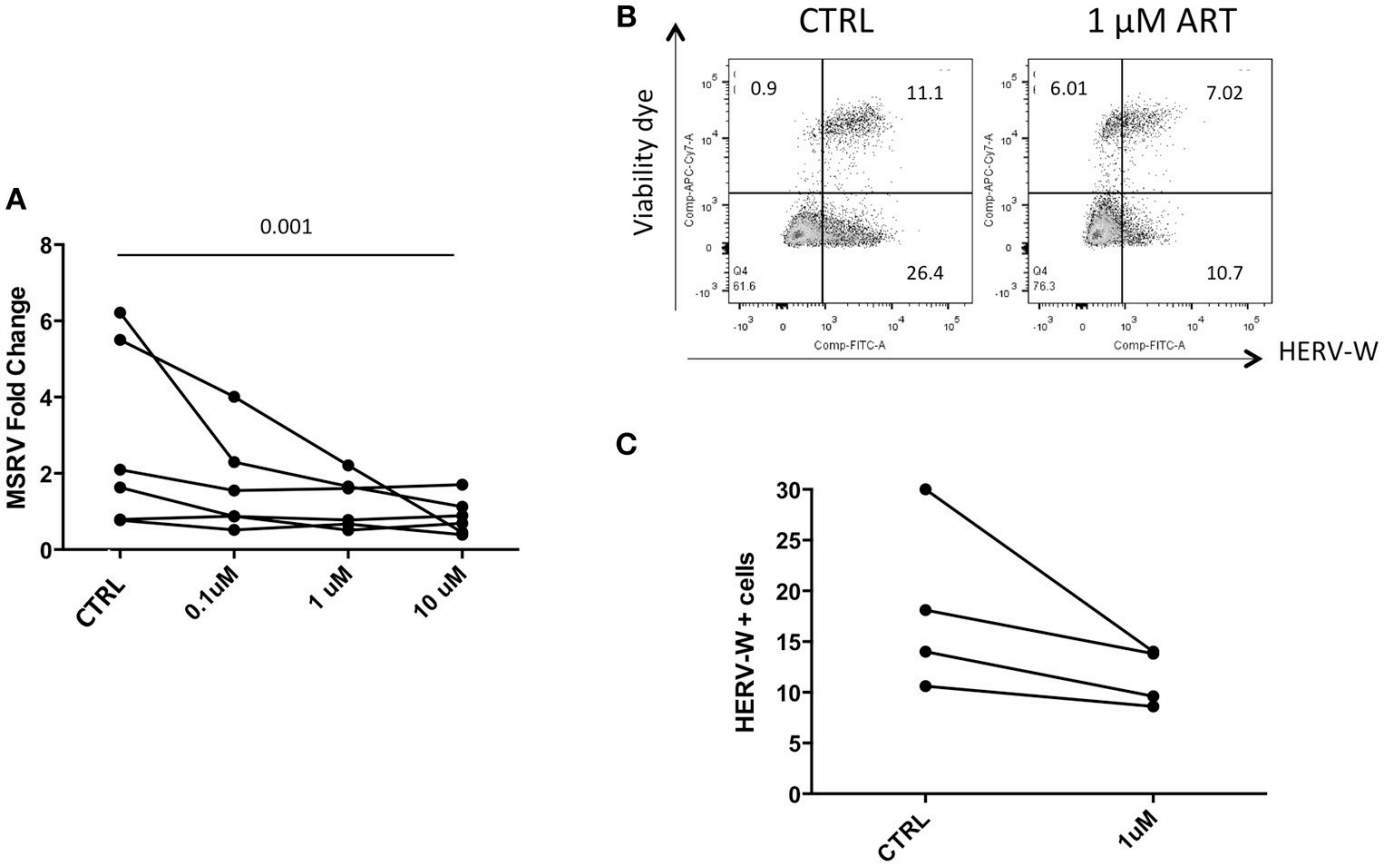
Combined ART decreases the expression of HERV-WEnv protein. **(A)** LCL from 6 different donors were treated with combined Lamivudine, Tenofovir, Daranuvir, Efavirenz, and Raltegravir at 10, 1, and 0.1 μM for 5 days. CTRL represents LCL treated only with DMSO. MSRV/HERV-W*env* expression was evaluated by relative quantification RT-PCR using the 2^−Δ*ΔCt*^ method. Each point represents an LCL (*n* = 6; Friedman test). **(B)** LCL were treated with combined drugs at 1 μM for 5 days. HERV-WEnv expression was evaluated by extracellular FC with dead cell marker included. One example is shown. **(C)** HERV-WEnv expression of 4 individual LCL is illustrated in dots before and after the treatment (*n* = 4; Wilcoxon test).

Cells were then treated with drug combinations, to mimic ART *in vivo*. Detection by RT-qPCR showed a reduced expression of MSRV/HERV-W*env* RNA (Figure 6A). Similarly, HERV-W/HERV-WEnv protein showed by FC a trend of reduction in the presence of 1 μM of combined drugs (Figures 6B,C).

## Discussion

The expression of MSRV/HERV-W*env* was evaluated in a small number of MS patients, HIV-infected patients, and HC. Expression of MSRV/HERV-W*env* in MS patients was higher compared to HC, as already reported in the literature (4). HERV-W Env was expressed in whole PBMC, at a high level in B cells and monocytes. No expression of HERV-W Env was detected in T cells. These results are in line with previous publications (23, 26) and underline a key role of B cells and monocytes in HERV-W expression. Despite increased expression of MSRV/HERV-W *env* mRNA in MS patients by RT-PCR, HERV-W Env protein was detected in all samples of MS and HC using FC with no difference in the number of HERV-W positive cells between the two groups. At the protein level, no antibody has been developed yet to discriminate MSRV Env from Syn-1 (18). Moreover, the issue of HERV-W complexity extends to studies of HERV protein expression: it often remains unclear as to which genomic locus the observed HERV proteins are derived from and therefore, whether the precise identity of the protein recognized by the HERV-W Env antibody can be established. This could explain why HERV-W is expressed in similar percentage in both groups: the antibody could detect either MSRVenv or Syn-1 or both together.

As reported in the literature, EBV infection induces the expression of MSRV/HERV-W *env* (25). However, the activation of the retrovirus can occur not only in the presence of EBV, but also when B cells are activated with CpG. Thus, bacterial or synthetic unmethylated CpG ODNs mediate their effects by interacting with TLR9, which is expressed in pDC and B cells. Activation of B cells by CpG leads to proliferation, production of antibodies and cytokines. It is possible therefore that MSRV/HERV-W activation is induced either directly by TLR9 activation as in the presence of a bacterial infection or by pro-inflammatory cytokines expressed by CpG-activated B cells. In line with this contention, it has been reported that pro-inflammatory stimuli, such as IFN-γ and TNF-α can activate MSRV/HERV-W *env* expression in B cells (23). MSRV/HERV-W *env* expression in LCL was not different from HC and people with MS, suggesting that EBV infection may potently induce expression of MSRV/HERV-W*env* and mask the original difference in MSRV/HERV-W *env* expression between B cells of HC and MS patients.

Expression of MSRV/HERV-W *env* in HIV-infected patients was similar to HC but was significantly lower than in MS patients. The relationship between exogenous and endogenous retroviruses is of relevance to human health and disease. All retroviruses have a similar genetic make-up, and homologous proteins encoded by one retrovirus could theoretically perform comparable functions for another member of the family and could thus complement a defective virus (27). Indeed, it has been reported that HIV, through its protein TAT, increases the expression of MSRV and HERV-K, as well as TLR4, in isolated B cells, NK cells and monocytes *in vitro* (28). A reduction of HERV-K expression in people who received suppressive ART has been reported (29, 30), but other HERV families have not been investigated. It is unclear whether HERV-K was directly inhibited by the antiretroviral drugs, or whether the loss of activation by HIV was responsible for the reduction of HERV-K viral load. It is possible that the inhibition of the RT in some way affects also the transcription of the HERV RNA. Although there is no evidence that HERV-W can function as a retrovirus capable of retro-transcription, some HERVs, and in particular HERV-K, can (31). Protease inhibitors targeted at HIV are not active against HERV-K (32), but other classes of antiretroviral drugs, in particular the ones that target the RT enzyme, might be. Interestingly, the non-nucleoside RT inhibitors Nevirapine and Efavirenz have already been shown to inhibit efficiently the normal endogenous RT activity that is detectable in many human cell lines and leukemic cells (33, 34), suggesting a possible efficacy of this drug class in the inhibition of HERVs.

The results of this pilot study are somewhat conflicting with the original hypothesis that ART would reduce the expression of HERVs, but our data are potentially explainable with consideration of the biology of the system. HIV patients did not show an increase in HERV-W expression when compared with healthy controls. This is possibly explained by a mismatch between the cell types that produce HERV-W (primarily monocytes and B cells) and those that are affected by HIV (primarily T cells). In this study, RNA was extracted from HIV patients from total peripheral blood and is not representative of a specific cell type. During HIV infection, there is an alteration in immune cell populations [e.g., with B cells there is hyper activation, as well as increased apoptosis that results in B cell exhaustion (35)]. Therefore, it is possible that in HIV patients MSRV/HERV-W *env* expression increases in some cell subpopulations, but this change might not detectable in whole blood. Moreover, RT-qPCR may not be the best method to detect MSRV expression (4, 36, 37).

When HIV patients were divided into ART-treated and untreated, no difference was found between the two groups. However, due to the low number of HIV patients not on ART treatment (*n* = 3), we cannot finally confirm that ART *in vivo* does not affect MSRV/HERV-W expression.

Analyzing the patients' HIV viral load, there was no correlation between HIV viral load and MSRV/HERV-W*env* expression (Table 1). It is possible that the different ethnicities of untreated HIV patients (Jamaican, Greek, and Filipino) and those treated (all white British) could influence the expression of MSRV/HERV-W *env* on the basis of genetic differences. MS patients in our studies were also mainly white British.

On the other hand, TLR4 expression was higher in both MS and HIV patients compared with HC. This is potentially due to the recognition of retroviruses, endogenous for MS and exogenous for HIV, as a viral antigen by the TLR system (7, 8). Indeed, it has been reported that myeloid dendritic cells from HIV patients had an increased expression of TLR2 and TLR4 compared to HC (37). Hernandez et al. also showed that the expression of TLR4 in cells from untreated patients was higher than in treated and that in monocytes there was a positive correlation between both the expression of TLR2/4 and viral load, but no correlation with CD4+ T cell numbers (38). In our study TLR2 was found to be increased only in MS patients, as already reported in the literature (39, 40), but not in HIV patients. This might be due to the increased MSRV/HERV-W *env* expression (6), or to other infections (39).

In a move to identify the *in vitro* effects of different concentrations of drugs used by the patients on LCLs, we have recorded that only 10 M Efavirenz (NNRTI) induced reductions in MSRV/HERV-W *env* expression. However, there was no clear evidence of inhibition in patients on NNRTI drugs as part of the ART mix treatment (Table 1).

When LCLs were treated with a combination of drugs a reduction of MSRV/HERV-W *env* RNA was detected (Figure 4A). This would suggest that a synergy between different drug classes is required to produce a measurable decrease in HERV-W expression, a contention which is in line with the demonstrable efficacy of ART against HIV as opposed to single drug therapy. Again, this effect was not seen in the HIV samples though this finding could have been accounted for by a) the mismatch between affected cells and total blood cell population described above; b) individual patient variability, and c) the small number of patients on each combination of drugs.

The number of HIV patients in this pilot study was too small to draw definite conclusions about the association of HIV with MSRV and the role of ART. It was particularly difficult to enroll untreated HIV patients. Until larger studies are conducted, we can at this stage only speculate about these mechanisms. Therefore, larger cohorts of HIV patients are much needed to recruit to identify whether ART has a role in protecting from MS.

In conclusion, HIV-infected patients had lower expression of MSRV/HERV *env* than MS patients, regardless of ART status. Among ARTs, Efavirenz may reduce the expression of HERVs, warranting further investigations to clarify the potential role of ART in protecting from MS.

## Ethics Statement

This study was carried out in accordance with the National Institute for Health Resaerch (NIHR) Good Clinical Practice Guidelines, with written informed consent from all subjects. All subjects gave written informed consent in accordance with the Declaration of Helsinki. The protocol was approved by the Nottingham Research Ethics Committee 2 (reference 08/H0408/167) and sponsored by the University of Nottingham.

## Author Contributions

EM conducted the experiments, analyzed the data and wrote the first draft of the paper. EM, RET, and BG designed the study. DC-T supervised the RT-PCR experiments. BG and RT provided blood samples from people with RRMS and age- and gender-matched healthy controls. All the authors critically edited the manuscript and BG and RET supervised the study.

### Conflict of Interest Statement

BG has received honoraria for consultancy from Merck, Roche, Biogen, and Teva UK and unrestricted research grants from Biogen Idec, Merck, Bayer Healthcare, Teva UK, Novartis, and Genzyme. None are related to this study. The remaining authors declare that the research was conducted in the absence of any commercial or financial relationships that could be construed as a potential conflict of interest.
